# A cognitive-motor intervention using a dance video game to enhance foot placement accuracy and gait under dual task conditions in older adults: a randomized controlled trial

**DOI:** 10.1186/1471-2318-12-74

**Published:** 2012-12-14

**Authors:** Giuseppe Pichierri, Kurt Murer, Eling D de Bruin

**Affiliations:** 1 Department Health Sciences and Technology (D-HEST), Institute of Human Movement Sciences and Sport, ETH Zurich, Zurich, Switzerland

**Keywords:** Dual task, Dance video game, Gait, Older adults, Cognitive-motor intervention

## Abstract

**Background:**

Computer-based interventions have demonstrated consistent positive effects on various physical abilities in older adults. This study aims to compare two training groups that achieve similar amounts of strength and balance exercise where one group receives an intervention that includes additional dance video gaming. The aim is to investigate the different effects of the training programs on physical and psychological parameters in older adults.

**Methods:**

Thirty-one participants (mean age ± SD: 86.2 ± 4.6 years), residents of two Swiss hostels for the aged, were randomly assigned to either the dance group (n = 15) or the control group (n = 16). The dance group absolved a twelve-week cognitive-motor exercise program twice weekly that comprised progressive strength and balance training supplemented with additional dance video gaming. The control group performed only the strength and balance exercises during this period. Outcome measures were foot placement accuracy, gait performance under single and dual task conditions, and falls efficacy.

**Results:**

After the intervention between-group comparison revealed significant differences for gait velocity (*U* = 26, *P* = .041, *r* = .45) and for single support time (*U* = 24, *P* = .029, *r* = .48) during the fast walking dual task condition in favor of the dance group. No significant between-group differences were observed either in the foot placement accuracy test or in falls efficacy.

**Conclusions:**

There was a significant interaction in favor of the dance video game group for improvements in step time. Significant improved fast walking performance under dual task conditions (velocity, double support time, step length) was observed for the dance video game group only. These findings suggest that in older adults a cognitive-motor intervention may result in more improved gait under dual task conditions in comparison to a traditional strength and balance exercise program.

**Trial registration:**

This trial has been registered under ISRCTN05350123 ( http://www.controlled-trials.com)

## Background

In the growing population of older people falling is a common problem. Approximately 30% of older adults over 65 years of age, experience a fall each year [1-3]. Fall incidence is even higher (50%) in women aged 85 and above [4]. Individuals who require more time to initiate and execute a step to avoid a threat or to recover postural balance, either during walking or performing postural transitions, may be at greater risk for falling [5]. Similarly, variable spatio-temporal gait characteristics may increase this risk [2]. For safe walking the accuracy with which one places the foot on the walking surface is essential, especially in challenging environments. Decreased foot placement accuracy [6,7] together with an increased variability in spatio-temporal gait characteristics [8], and dual task deficits [9] are typical symptoms of the ageing process and constitute critical factors that compromise safe walking [10].

Common tasks of daily life such as walking are dependent on both sensorimotor processes and higher level cognitive functions [9]. Thus, the accurate foot placement onto a footpath for instance, requires not only the appropriate planning and execution of the movement. It also requires visual scanning, the extraction of visual information from the environment, and cognitive skills related to so-called executive functioning processes [7,11,12]. The term executive functioning processes refers to a group of cognitive actions that include: dealing with novelty, planning and implementing strategies for performance, using feedback to adjust future responding, vigilance, and inhibiting task-irrelevant information [13]. There also is increasing evidence that an age-related decline in visuo-motor control contributes to deficiencies in foot placement control [14]. Suboptimal visual sampling strategies have been observed in older adults prone to falling when taught to step into a target location [7,14,15].

Exercise interventions that incorporate exercises to improve muscle strength and postural control have been often recommended for older adults [16]. However, with physical exercise alone the additional cognitive requirements of safe walking cannot be addressed. Two recent reviews discussed the interplay between physical functions and cognition [17,18]. Both brought out the importance to combine physical and cognitive training into clinical practice to enable older adults to move safer in their physical environment. Especially computerized interventions seem to be promising for this purpose [17]. Thus, cognitive elements should be taken into account when designing an exercise regimen with the aim to preserve or improve walking skills in older adults [19,20]. Interventions should thereby focus on executive functioning processes [19], in particular on divided attention [21], and should provide physical activities with decision-making opportunities because these are believed to be able to facilitate the development of both physical performance and brain functions [22].

A simple and motivating way to incorporate a cognitive element into a physical exercise program is the use of interactive video games. Interactive video games seem to have the potential to train cognitive functions [23] such as executive functioning processes [24]. An interactive dance video game for example [25-27], requires the player to observe the virtual environment for drifting cues and to concurrently execute well-coordinated body movements, thus, challenging in particular divided attention skills. Previous dance video games studies have shown the feasibility, defined through recruitment, attrition and adherence to the exercise intervention [28], of this approach. Dance video games studies in senior living settings [26,27,29,30] or with post-menopausal women [31] have also shown that this approach is a safe, low-cost and motivating way to activate and ensure continuation of physical exercise in middle-aged and older adults. Further, positive contributions to self-reported balance confidence and mental health were observed [26,29]. The results of two pilot studies conducted in care home settings, have shown that the addition of dance video gaming may have a positive effect on relative dual task costs of walking [30] and gait initiation under attention demanding circumstances [27] even in the oldest old (85 years and beyond). These latter two findings, however, should be interpreted with caution. In both studies the control group did not train but rather underwent usual care. To clarify the additional influence of the dance video game we should consider a study design with a control group performing the same strength and balance exercises, however, without the additional dance video gaming [27].

This study compares two training groups that achieve similar amounts of physical strength and balance exercise, where one group additionally performs dance video game training. The aim is to investigate the additional effects of the dance video game training on foot placement accuracy [7,15], gait under single and dual task conditions, and on fear of falling.

## Methods

### Participants

The study was designed as a prospective randomized controlled trial (ISRCTN05350123) and was carried out from June to September 2011. Participants were recruited from two hostels for the aged in the Canton of Zurich, Switzerland. The study protocol was approved by the local ethics committee (KEK-ZH-NR 2011-0005/0). All measurements and trainings were performed in suitable locations at the hostels.

The residents of both hostels were invited to attend an information session. Thirty-five (67.3%) out of 52 persons were interested in participating and were assessed for eligibility. Participants were included if they were older than 65 years, had a score of at least 22 points on the Mini-Mental State Examination (MMSE) [32], were able to walk for at least eight meters with or without the need for a walking aid, and were free of rapidly progressive or terminal illness, acute illness or unstable chronic illness. If unsure, subjects were asked to consult their primary care physician for medical clearance. They were excluded if a severe impairment of vision would impede to see projections on a wall screen as needed for the intervention.

Four interested persons were excluded from the study before the randomization process. One person changed his mind and declined to participate due to insufficient motivation. One person suffered from hernia inguinalis before the start of the study and two other persons were excluded for not fulfilling the inclusion criteria (MMSE < 22). A total of 31 (59.6%) eligible residents signed informed consent statements and were randomly assigned to either the ‘Dance group’ (DG, n = 15) or the ‘Control group’ (CG, n = 16) using a random numbers table. Blinding of investigators was not possible because the investigators supervised and conducted the training sessions.

### Intervention

The DG and the CG underwent a twice weekly physical exercise program consisting of progressive resistance and postural balance training for twelve weeks. Intensity and duration of the program were chosen based on the guidelines published by the American College of Sports Medicine [33,34] and on a review by Paterson et al. describing exercise recommendations for older adults [35]. Training sessions were conducted in groups of three or four participants to form group cohesion and to encourage exercise class participation [36]. A training session lasted on average 40 minutes and consisted of a warm-up (5 minutes), resistance training (25 minutes), and balance exercises (10 minutes).

In addition to the physical exercise program the DG performed a progressive video game dancing program for 10–15 minutes throughout the study (‘cognitive-motor program’).

**Figure 1:**
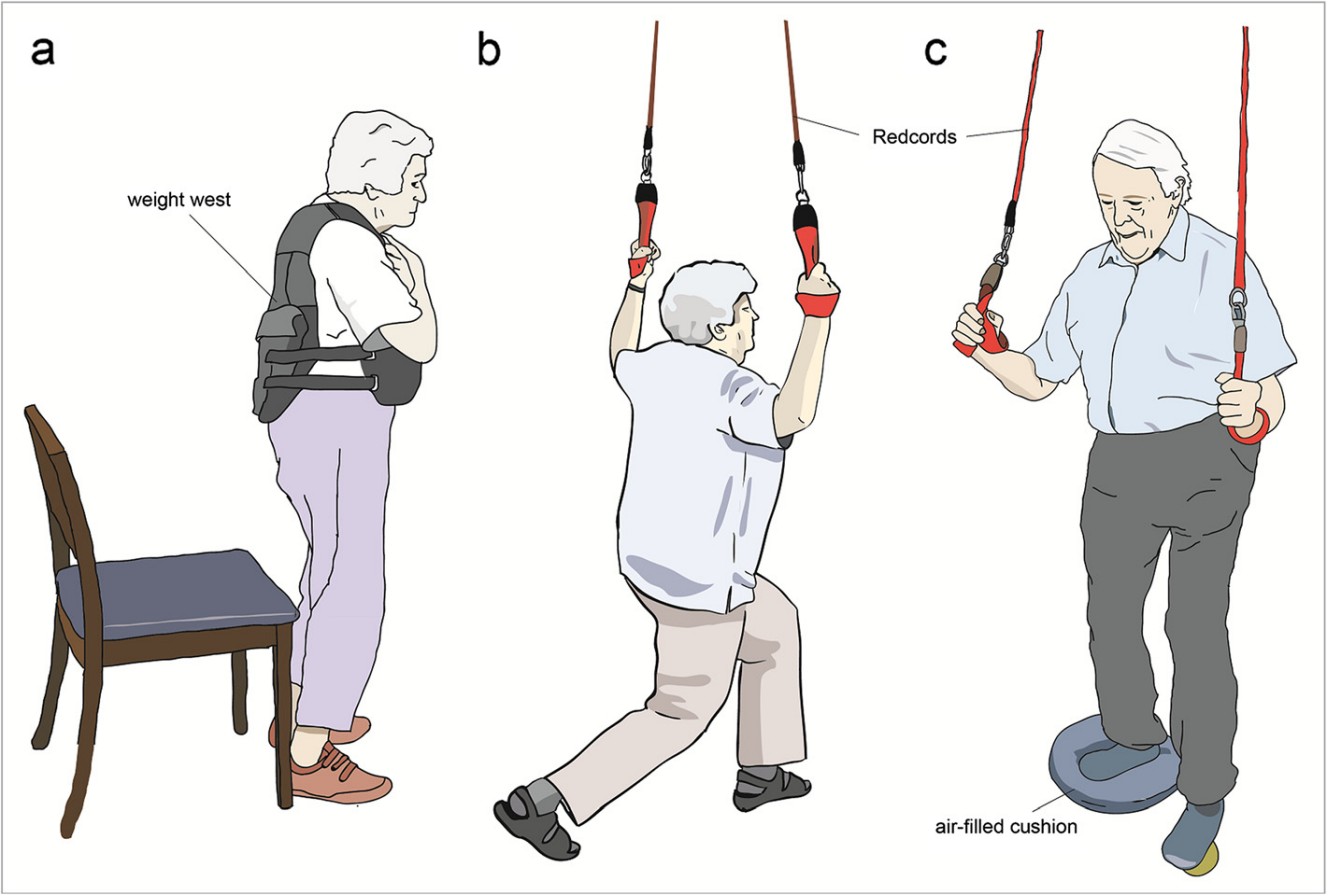
Exercise examples from the physical exercise program: strength exercises (a) sit-to-stand, (b) squat; (c) balance exercise: subject rolls the ball back and forth or from the left to the right with the left foot while balancing on the air-filled cushion.

**Figure 2:**
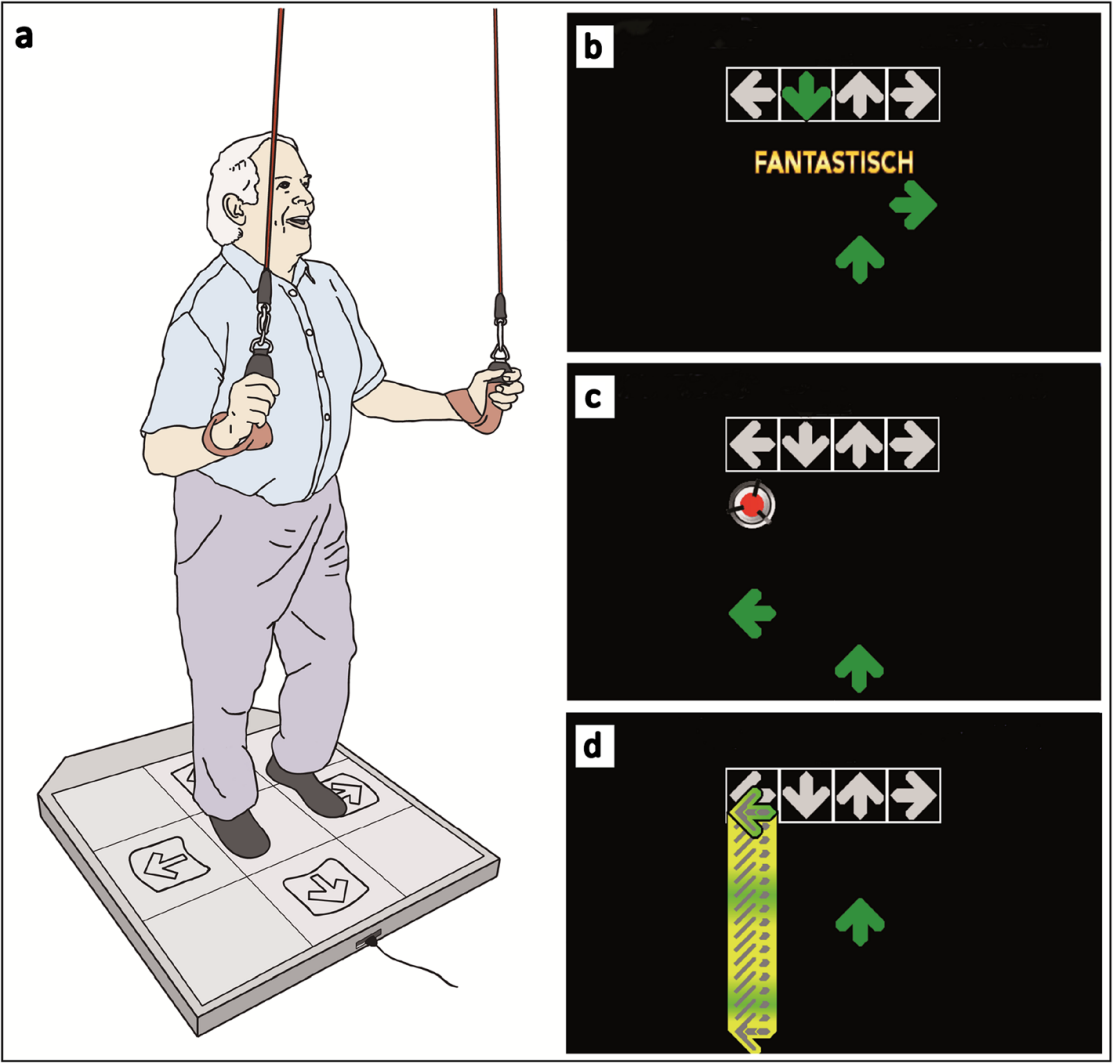
Dance video game: (a) participant on the dance pad secured by ropes fixed on the ceiling; (b-d) screenshots of the dance video game.

When exercises required the participants to stand, they were requested to hold on to ropes fixed on the ceiling for safety reasons (Figures 1 and 2) (Redcord AS, Staubo, Norway).

#### Physical exercise

The progressive resistance training focused on the muscle groups of the core and lower extremities that are used in functional activities of daily living (Figure 1a-b). Two sets of ten to 15 repetitions of each exercise in a slow, controlled manner were performed. One minute sitting breaks after each set and between the series were provided. Training intensity was controlled by perceived exertion and intensity between “somewhat hard” and “hard (heavy)” on Borg’s perceived exertion scale [37]. To maintain the intensity of the stimulus during the training period, the number of repetitions and the load were progressively increased with weight vests (Kettler GmbH & Co. KG, D-59469 Ense-Parsit), as tolerated by the participants.

The progressive postural balance program (Figure 1c) consisted of static and dynamic functional balance exercises using air-filled cushions and grip balls (Ledraplastic S.p.a, I-33010 Osoppo) [38]. A more detailed description of the program can be found elsewhere [38].

#### Cognitive-motor program

As an additional cognitive element the DG performed the dance video game after the physical exercise in every training session. The dance video game was performed on metal dance pads (Figure 2a) (TX 6000 Metal DDR Platinum Pro, 93 x 14.7 x 109 cm, Mayflash Limited, Baoan Shenzhen, China) and with a specially designed modification of the StepMania (Version 3.9) free-ware [27,30]. The dance video game screen was projected on a white wall. A scrolling display of arrows moving upwards across the screen cued each move, and the participants were asked to execute the indicated steps (forward, backward, right, or left) when the arrows reached the fixed raster graphic at the top of the screen (Figure 2b-d), and in time with different songs (32 to 137 beats per minute). In the first training session a tutorial sequence was provided to ensure understanding of the task. As the levels increased additional distracting visual cues, e.g., “bombs,” were presented (Figure 2c). Participants had to ignore these cues and keep their attention focused on the arrows. Occasionally, some arrows were drawn-out on the target locations indicating that the trainees should remain for a while on the dance pad button on one leg (Figure 2d). The arrow sequences were generated using the Dancing Monkey MATLAB script [39]. Electronic sensors in the dance pad detected position and timing information that was then used to provide participants with real-time visual feedback. For each training session, the participants performed for four songs of two to three minutes length each, with a short break of 30 seconds after each song. Progression of performance was controlled through the beats per minute and the difficulty level.

### Baseline assessments of vision

To ensure that participants were free of vision impairments, which would have complicated the performance of the video game dancing part, participants’ vision skills were assessed with the vision tests of the ‘Physiological Profile Assessment’ (PPA) [40,41]. Tests of edge contrast sensitivity (‘Melbourne Edge Test’), and binocular dual-contrast visual acuity, were considered for assessing vision.

### Test procedures and outcomes

The following tests were performed in the week before and in the week after the twelve weeks training period in suitable locations at the hostels.

#### Foot placement accuracy test

**Figure 3:**
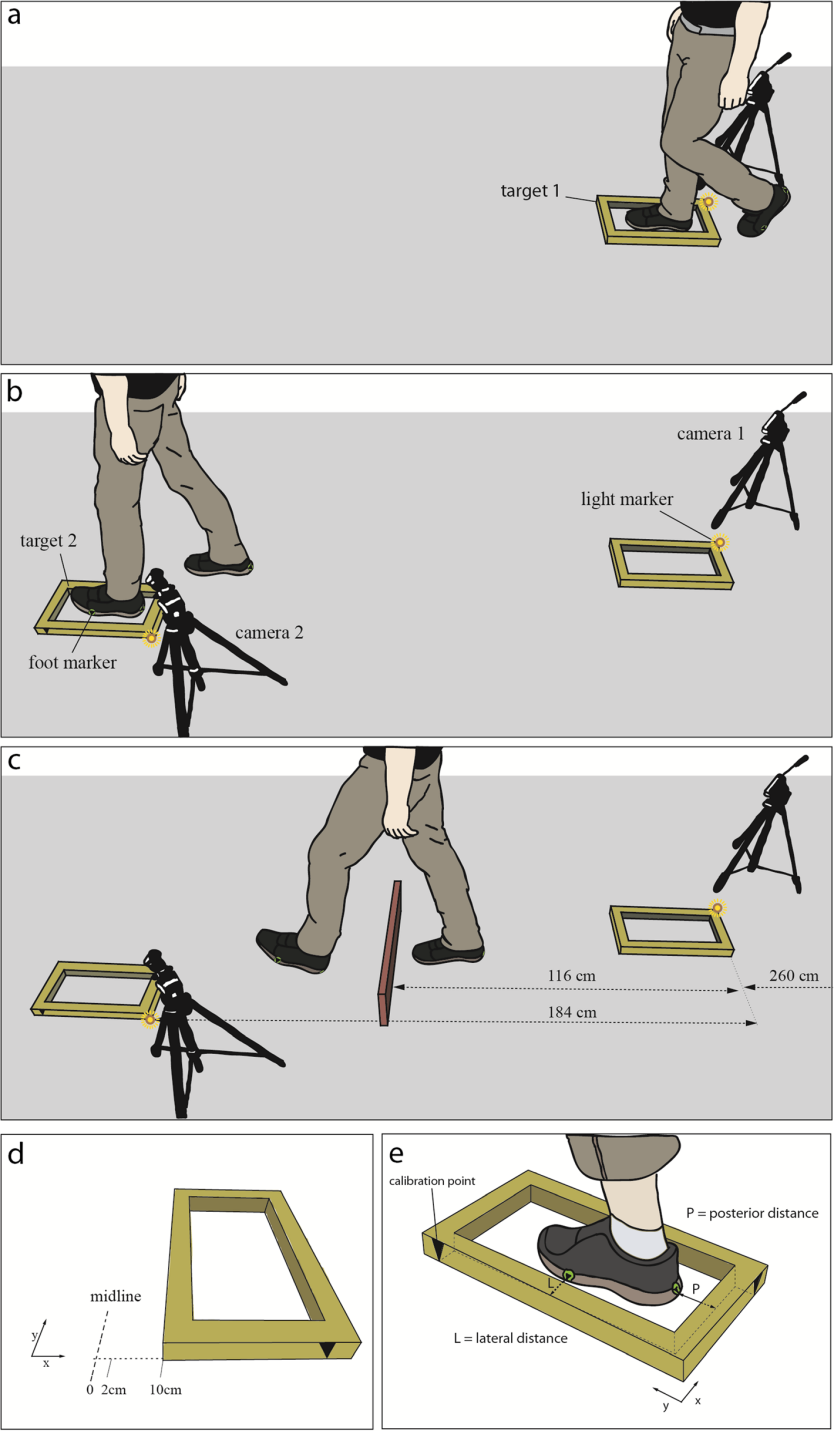
**Set-up of the foot placement accuracy protocol.** Subjects were required to walk along the pathway at self-selected pace and place the right foot into target 1 (Condition 1 (**a**)), place the right foot into target 1 and the left foot into target 2 (Condition 2 (**b**)), and additionally step over an obstacle lying between the two targets (Condition 3 (**c**)). The target(s) appeared in two possible positions separated medio-laterally by 8 cm to prevent task familiarization (**d**). Video still of camera 2 during stance phase used to evaluate the lateral and posterior distance error (**e**).

Foot placement accuracy (FPA) was assessed with an adapted version of the protocol described by Chapman and Hollands in 2007 [7]. Subjects were instructed to walk at self-selected walking speed along a path with three different walking conditions: ‘Condition 1’ required placing the right foot into Target 1 (T1) (Figure 3a); in ‘Condition 2’ subjects placed the right foot into T1 and the left foot into Target 2 (T2) (Figure 3b); ‘Condition 3’ additionally required stepping over an obstacle placed between the two targets (Figure 3c). The targets and the obstacle were made of soft foam material. The targets were rectangular (target area: 190 mm x 415 mm) and comprised a raised border (40 mm x 40 mm x 40 mm). Dimensions of the obstacle were 170 mm x 670 mm x 25 mm (height x length x depth). 170 mm corresponds to the ideal height of a staircase step as defined by the Federal Authorities of the Swiss Confederation. The subjects performed ten repetitions for each of the three walking conditions, resulting in a total of 30 trials per person. To prevent task familiarization within each condition, the target(s) appeared in two possible positions separated medio-laterally by 8 cm (Figure 3d), an adaptation to the protocol based on the results of Young and Hollands [15]. Prior to the start of each trial subjects stood with their back against the walking path facing a wall to limit the amount of attention towards the pathway during the adjustments of the target position by the investigator. After a light cue, triggered by the investigator, subjects turned towards the walking path and began to walk from a labeled starting position over the path at a self-selected pace. Subjects were verbally instructed prior to each change of walking condition to place their foot as accurately as possible in the middle of the target area and were allowed to perform two rehearsal walks for each condition. The presentation of the target positions was randomized and of equal number for each condition. The presentation of the walking conditions was not randomized.

To assess participants’ foot placement performance into the targets, adhesive labels were placed on both shoes on calcaneus level for anterior-posterior (A-P) distance assessment and on the level of the head of the fifth metatarsal for medio-lateral (M-L) assessment (Figure 3e). During each trial the targets were recorded by two stationary video cameras (Contour HD 1080p) at a sampling rate of 30 Hz. A fixed image of the foot’s stance phase into the target was then read into the analyzing software (Vicon Motus 9.2, Vicon Motion Systems, Oxford, UK). The foam targets were previously marked with two triangles (Figure 3e), serving as calibration points to read in the dimensions and to determine spatial orientation of the target by the video analysis software.

The main outcome of the FPA test was the M-L and A-P deviation in mm of the foot center to the center of the foam target. Further, walking velocity between T1 and T2 and quality aspects of the performance during the FPA test were assessed. For the quality evaluation the number and percentage of contacts of the leading or the subsequent foot with the target, and the use of the wrong foot were assessed.

#### Gait analysis

Spatio-temporal gait parameters were assessed with GAITRite^®^ Platinum Version 4.0 software and the GAITRite^®^ electronic walkway (CIR Systems, Havertown, USA) with a sampling rate of 60 Hz [42-44]. The GAITRite® with an active area of 7.92 meters length was extended with two 2.5 meters carpets at the beginning and at the end to eliminate the effects of acceleration or deceleration and to allow for steady state gait assessment. Subjects were instructed to walk over the electronic walkway under four different conditions: at self-selected comfortable walking speed (*normal*) and at a fast walking speed (*fast*, as fast as possible without running) each with or without a concurrent cognitive task (*normalcog* and *fastcog*), respectively. The additional cognitive task consisted in counting out loud backwards by steps of seven from a three-digit number given by the investigator at the start of each trial. For each walking condition three trials were collected, resulting in twelve walks per participant. Subjects were allowed to wear their everyday footwear.

The temporal-spatial parameters recorded were: velocity (cm/s), cadence (steps/min), step time (s), cycle time (s), stance time (s), single support time (s), double support time (s), and step length (cm). Relative dual task costs (DTC) of walking were calculated as percentage of loss relative to the single-task walking performance, according to the formula DTC [%] = 100 * (single-task score - dual-task score)/single-task score [45]. The effective DTC changes were defined as the mean difference between pre and post intervention (Δ DTC).

#### Gaze behavior

ASL Mobile Eye, a head-mounted eye-tracking system (Applied Science Laboratories, Bedford, USA) was used to assess gaze behavior during the FPA test. Based on the observation that older adults prone to falling look away from a target location prior to heel contact on the floor [7], the temporal-spatial gaze parameters were defined as: a) location of gaze at heel contact (on/off target), b) the subsequent duration (milliseconds) of target fixation after heel contact, or in case of a premature gaze shift c) the time elapsed between the early gaze shift away from target and the heel contact (milliseconds). Gaze fixation was defined as a stabilization of gaze in the environment for longer than 120 milliseconds [7]. The light cue, serving as a start signal for the FPA test, was filmed by the eye-tracking camera and served as a marker for the synchronization of the eye-tracking camera with the stationary cameras pointed towards the targets.

#### Fear of falling

The Falls Efficacy Scale International (FES-I) questionnaire was used as a measure of concern about falling to determine the transfer effects of training to activities of daily living [46].

### Statistical methods

A 75% attendance rate for the training sessions was set as the definition for being adherent to the training program [47]. There were a total of 24 training sessions scheduled for each individual in the study. Only those subjects who adhered to the training counted towards the final results (per protocol analysis).

An average value of the M-L and A-P distance errors for each walking condition and target in the FPA test, as well as for each spatio-temporal gait parameter of the gait analysis for each walking condition, was calculated. Due to non-normality of the data a comparison at baseline was undertaken using a Mann–Whitney-*U*-test. The Mann–Whitney-*U*-test was also used to estimate group interaction effects (between-groups differences), after the twelve-week training period. For this purpose the difference of the values pre and post intervention for each subject were calculated and then compared. The effects size, *r*, was calculated as $r\;=\;\frac{Z}{\sqrt{N}}$ (where Z is the approximation of the observed difference in terms of the standard normal distribution and N is the total number of samples; *r* = 0.1, small effect; *r* = 0.3, medium effect; and *r* = 0.5, large effect). A Wilcoxon signed rank test was used to compare within-group pre/post data. The significance level was set at *P* ≤ 0.05. A trend to significance was defined as 0.05 < *P* ≤ 0.10. Statistical computations were carried out with SPSS 19.

## Results

**Figure 4:**
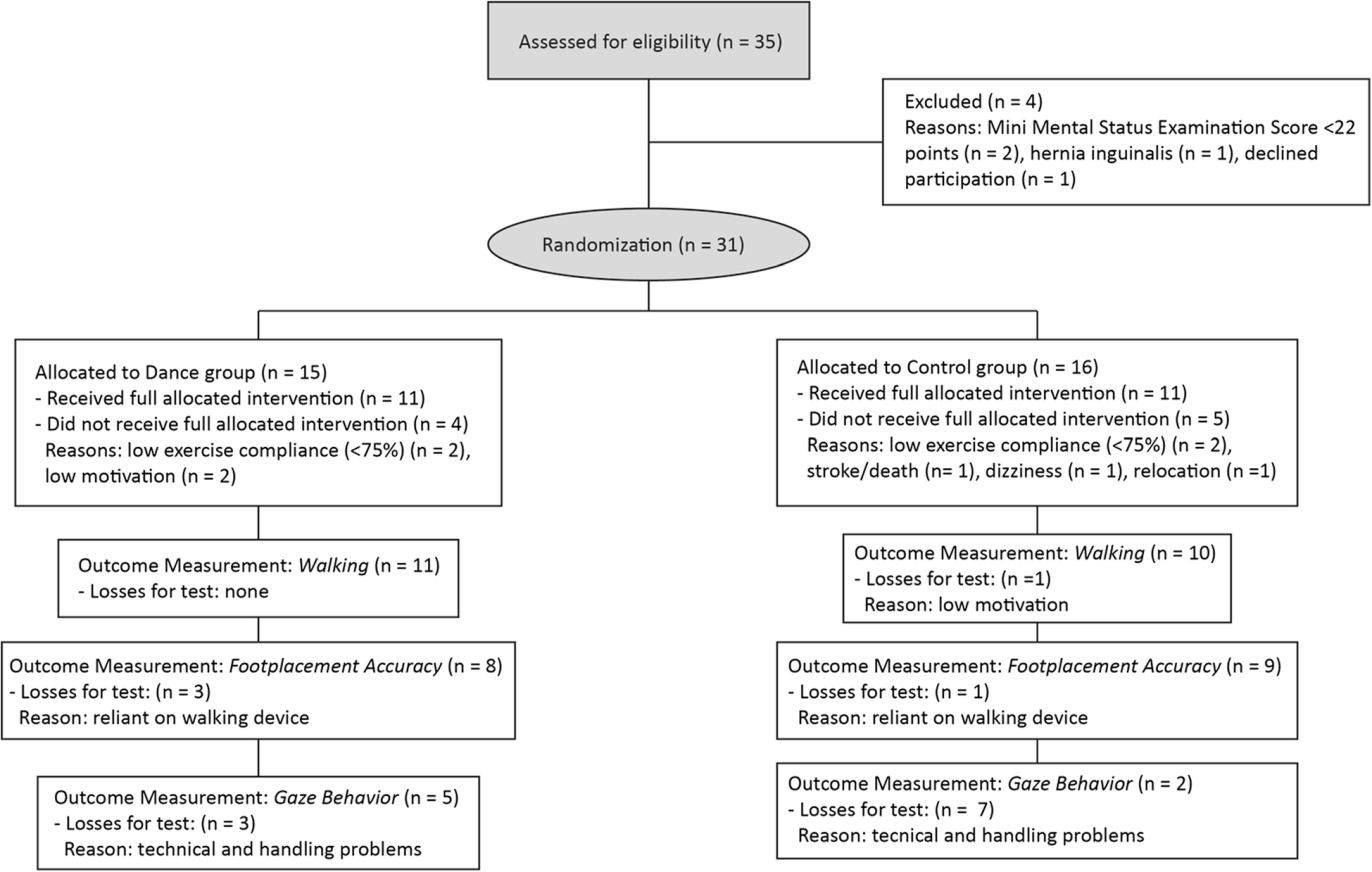
The study flow chart.

A total of 22 participants (mean ± SD) age: 86.2 ± 4.6 years) received the full allocated intervention. Detailed information on subjects’ recruitment and reasons for loss are presented in the flow chart (Figure 4). Table 1 shows demographic and clinical characteristics of the sample. Eleven (50%) subjects were classified as having a high risk for falling based on the presence of at least four of eight possible risk factors according to di Fabio et al. [48]. No significant differences at baseline, neither in demographic nor in the outcome measurements, were observed between the groups. No subject manifested a severe impairment of vision.

**Table 1:** Demographic baseline data of participants

**Group**	**Dance group**	**Control group**
No. of participants	11	11
Age (mean, SD)	86.9, 5.1	85.6, 4.2
Sex (female/male)	8/3	10/1
Height in cm (mean, SD)	159.1, 10.0	154.5, 10.1
Weight in kg (mean, SD)	68.3, 14.3	58.8, 13.1
Mini-Mental Status^a^ (mean, SD)	27.2, 2.0	27.0, 2.6
***Vision***		
Vision aid (n/%)	5/45.4	4/36.4
Visual acuity^b^ [MAR] (high/low contrast)	0.48/0.66	0.53/0.67
Melbourne Edge Test^c^ [dB] (mode, range)	19, 9-21	19, 5-24
***Fall Risk factors***^d^***(n/%)***		
Low BMI (< 23)	1/9	5/4
Slow walking speed (< 1.22 m/s)	11/100	11/100
Previous falls requiring medical attention	none	none
Falls in the last 6 months	1/9	3/27
3 or more prescription medications	7/64	10/91
Cardiovascular medications	11/100	10/91
Anti-Anxiety medication or sedatives	3/27	3/27
Medication for dizziness	none	1/9
Categorized as ‘Faller’	5/45	6/55

**Notes**: ^a^ = Minimum score = 0, maximum score = 30 (higher scores indicate better cognitive functioning); ^b^ log_10_ of the minimum angle resolvable (MAR) in minutes of arc (−log_10_(distance (3 m)/lowest correct line)); ^c^ = Minimum score = 1, maximum score = 24 (higher scores indicate better edge contrast sensitivity, 1 dB = −10 log_10_); ^d^ = Di Fabio et al. 2001 [48].

A fall was defined as an event, which results in a person coming to rest on the ground or other lower level.

**Abbreviations:***SD*, standard deviation; *arcmin*, arcminute.

An average exercise compliance of 90.6% (21.7 out of 24 sessions) was observed. The DG showed a compliance of 94.7% (22.7 out of 24 sessions) whereas the CG visited 86.9% of the exercise sessions (20.8 out of 24 sessions).

### Foot placement accuracy

A summary of the FPA test is provided in Table 2. A more detailed illustration of the FPA test data with the single target locations and conditions is provided in Additional file 1. The data of 17 participants were collected.

**Table 2:** Results of foot placement accuracy

	**Dance group (n = 8)**		***P***_***within***_	**Control group (n = 9)**		***P***_***within***_	***P***_**between**_
	**pre**	**post**		**pre**	**post**		
*Distance errors [mm]*							
Medio-lateral error	13.4 (10.0; 15.3)	10.1 (8.8; 11.95)	0.05*	12.9 (9.9; 16.2)	10.1 (8.7; 15.7)	*0.44*	*0.70*
Anterior-posterior error	16.6 (14.9; 29.2)	21.2 (15.1; 28.7)	*0.33*	21.7 (17.2; 26.9)	24.4 (15.5; 30.7)	*0.17*	*0.63*
*Walking velocity [cm/s]*							
Condition 2	53.0 (45.0; 67.0)	62.0 (57.0; 75.0)	0.02*	53.0 (41.0; 68.0)	49.0 (34.5; 57.5)	*0.81*	0.03*
Condition 3	42.0 (34.0; 43.0)	47.0 (45.0; 49.0)	*0.31*	33.0 (25.5; 59.5)	41.0 (24.0; 64.0)	*0.10*	*1.00*
*Quality evaluation (n, %)*							
Contact with leading foot							
medio-lateral	11 (2.8)	3 (0.8)		5 (1.1)	6 (1.3)		
anterior-posterior	17 (4.3)	8 (2.0)		21 (4.7)	10 (2.2)		
Contact with subsequent foot	16 (4.0)	14 (3.5)		14 (3.1)	16 (3.6)		
Wrong foot	2 (0.5)	2 (0.5)		5 (1.1)	2 (0.4)		

**Notes:** Distance error (absolute values) and walking velocity values are displayed as group medians with interquartile ranges (*q1; q3*) due to non-normal distribution of data; * = significant within-group differences pre-post (*P*_*within*_ ≤ 0.05) calculated with Wilcoxon signed rank test; percentage values are defined as the quotient between number of contacts or wrong foot, respectively, divided by the sum of trials of the whole group.

Abbreviations: *P*_*within*_: *P*-value for within-group comparison; *P*_between_: *P*-value for between-groups comparison.

Between-group comparisons (all conditions and targets) resulted in no significant differences of foot placement performance. Within-group comparison resulted in a significant improvement in M-L foot placement performance (*Z* = −1.960, *P* = 0.050) in DG and no changes in the CG.

The detailed results of the FPA test performance in A-P directions demonstrate an increase in distance error for both the DG and CG. In Condition 3, for the CG even a significant increase in A-P distance error from 8.34 to 18.75 mm was observed (*Z* = −2.100, *P* = 0.036).

Median walking velocity during the FPA test significantly increased in the DG in ‘Condition 2’ from 53.0 to 62.0 cm/s (*Z* = −2.371, *P* = 0.018), and the between-group comparison revealed significant differences in favor of the DG (*U* = −2.122, *P* = 0.034, *r* = 0.51).

### Gait analysis

The data of 21 subjects were collected for the gait analysis. The detailed results of the spatio-temporal gait analysis are summarized in Tables 3 and 4. Significant between-group differences where observed in the *fastcog* condition, where participants were required to walk as fast as possible with a concurrent cognitive task. The DG showed a significant increase in walking velocity (*U* = 26, *P* = 0.041, *r* = 0.45), and a decrease in single support time (*U* = 24, *P* = 0.029, *r* = 0.48) compared to the CG. The within-group comparison revealed significant walking performance improvements throughout all the walking conditions for the DG. In contrast, in the CG improvements in walking performance were only observable for the *normal* and *normalcog* conditions.

**Table 3:** Results of spatio-temporal gait analysis

**Condition/Parameters**	**Dance Group (n = 11)**		***P***_***within***_	**Control Group (n = 10)**		***P***_***within***_	***P***_**between**_	***r***
	**pre**	**post**		**pre**	**post**			
***normal***								
Velocity [cm/s]	80.4 (72.9; 89.1)	88.3 (69.2; 106.2)	*0.248*	69.0 (61.1; 82.7)	82.2 (73.8; 101.8)	*0.059*°	*0.481*	0.15
Cadence [steps/min]	95.5 (93.3; 102.5)	97.5 (96.0; 111.8)	0.016^*^	93.9 (80.2; 99.9)	104.2 (89.9; 112.3)	0.022^*^	*0.260*	0.25
Step time [s]	0.63 (0.59; 0.64)	0.62 (0.54; 0.63)	0.016^*^	0.64 (0.60; 0.75)	0.58 (0.54; 0.67)	0.022^*^	*0.205*	0.28
Cycle time [s]	1.26 (1.17; 1.29)	1.23 (1.07; 1.24)	0.016^*^	1.28 (1.20; 1.49)	1.15 (1.07; 1.34)	0.022^*^	*0.217*	0.27
Stance time [s]	0.82 (0.80; 0.86)	0.78 (0.72; 0.84)	0.021^*^	0.86 (0.78; 1.03)	0.75 (0.70; 0.92)	0.037^*^	*0.191*	0.29
Single support time [s]	0.42 (0.39; 0.44)	0.41 (0.36; 0.43)	0.041^*^	0.43 (0.41; 0.47)	0.39 (0.36; 0.43)	0.013^*^	*0.095*°	0.36^+^
Double support time [s]	0.41 (0.38; 0.43)	0.37 (0.31; 0.43)	*0.062*°	0.44 (0.37; 0.56)	0.36 (0.32; 0.49)	0.047^*^	*0.259*	0.25
Step length [cm]	50.7 (45.7; 53.0)	52.7 (42.6; 56.3)	*0.477*	46.6 (43.7; 52.2)	48.8 (46.0; 54.9)	*0.203*	*0.725*	0.08
***fast***								
Velocity [cm/s]	124.7 (89.9; 147.4)	132.7 (93.2; 149.8)	*0.091*°	112.1 (99.0; 124.0)	114.7 (99.6; 136.8)	*0.285*	*0.778*	0.06
Cadence [steps/min]	122.6 (113.8; 128.7)	127.1 (118.2; 134.5)	0.033^*^	118.2 (106.0; 129.1)	126.0 (105.7; 135.7)	*0.203*	*0.888*	0.03
Step time [s]	0.49 (0.47; 0.53)	0.47 (0.45; 0.51)	0.050^*^	0.51 (0.46; 0.57)	0.48 (0.44; 0.57)	*0.139*	*0.972*	0.01
Cycle time [s]	0.98 (0.93; 1.05)	0.94 (0.89; 1.02)	*0.062*	1.02 (0.93; 1.13)	0.96 (0.88; 1.13)	*0.139*	*0.972*	0.01
Stance time [s]	0.62 (0.58; 071)	0.60 (0.57; 0.66)	0.041^*^	0.65 (0.61; 0.72)	0.60 (0.55; 0.74)	*0.169*	*0.944*	0.02
Single support time [s]	0.37 (0.34; 0.38)	0.35 (0.32; 0.39)	*0.110*	0.38 (0.33; 0.39)	0.36 (0.33; 0.38)	*0.169*	*0.669*	0.09
Double support time [s]	0.25 (0.23, 0.33)	0.26 (0.22; 0.31)	*0.131*	0.30 (0.25; 0.32)	0.24 (0.22; 0.34)	*0.203*	*0.804*	0.05
Step length [cm]	58.4 (53.1; 66.7)	59.2 (50.5; 69.8)	*0.594*	56.0 (52.1; 61.0)	55.3 (50.4; 62.6)	*0.575*	*0.549*	0.13
***normalcog***								
Velocity [cm/s]	65.4 (57.3; 87.4)	82.8 (60.0; 97.9)	0.050^*^	60.0 (51.7; 71.8)	63.2 (55.8; 84.4)	0.028^*^	*0.673*	0.09
Cadence [steps/min]	87.0 (82.4, 93.8)	101.0 (97.2; 103.3)	0.026^*^	82.7 (73.9; 100.9)	96.8 (74.5; 103.6)	*0.139*	*0.481*	0.15
Step time [s]	0.69 (0.64; 0.73)	0.60 (0.58; 0.62)	0.013^*^	0.73 (0.60; 0.84)	0.62 (0.58; 0.81)	*0.074*°	*0.888*	0.03
Cycle time [s]	1.39 (1.28; 1.45)	1.19 (1.17; 1.24)	0.013^*^	1.46 (1.19; 1.64)	1.24 (1.16; 1.61)	*0.093*°	*0.573*	0.12
Stance time [s]	0.94 (0.85; 0.99)	0.79 (0.75; 0.83)	0.010^*^	1.00 (0.78; 1.15)	0.81 (0.79; 1.08)	*0.059*°	*0.672*	0.09
Single support time [s]	0.45 (0.43; 0.47)	0.41 (0.39; 0.44)	0.013^*^	0.46 (0.40; 0.48)	0.43 (0.38; 0.50)	*0.445*	*0.228*	0.26
Double support time [s]	0.48 (0.41; 0.55)	0.39 (0.35; 0.41)	0.016^*^	0.55 (0.39; 0.67)	0.43 (0.39; 0.59)	*0.059*°	*0.888*	0.03
Step length [cm]	46.73 (40.3; 51.1)	50.5 (44.3; 56.6)	*0.131*	43.4 (37.5; 52.4)	46.3 (38.1; 54.6)	*0.059*°	*0.751*	0.07
***fastcog***								
Velocity [cm/s]	87.9 (68.6; 103.2)	107.5 (74.1; 115.6)	0.013^*^	80.1 (61.0; 100.0)	87.1 (59.0; 103.2)	*0.386*	0.041^*^	0.45^+^
Cadence [steps/min]	99.1 (94.8; 105.5)	109.7 (101.7; 117.4)	0.006^*^	91.8 (88.0; 108.8)	104.1 (76.7; 114.1)	*0.445*	*0.057*°	0.42^+^
Step time [s]	0.61 (0.57; 0.63)	0.55 (0.51, 0.59)	0.006^*^	0.65 (0.55; 0.68)	0.58 (0.53; 0.78)	*0.799*	*0.062*°	0.41^+^
Cycle time [s]	1.21 (1.14; 1.26)	1.09 (1.02; 1.19)	0.006^*^	1.31 (1.10; 1.37)	1.15 (1.05; 1.57)	*0.799*	*0.091*°	0.37^+^
Stance time [s]	0.78 (0.71, 0.84)	0.71 (0.64; 0.78)	0.008^*^	0.86 (0.72; 0.93)	0.76 (0.70; 1.03)	*0.721*	*0.121*	0.34^+^
Single support time [s]	0.43 (0.39; 0.43)	0.38 (0.37; 0.41)	0.006^*^	0.42 (0.38, 0.46)	0.39 (0.37; 0.50)	*0.799*	0.029^*^	0.48^+^
Double support time [s]	0.38 (0.32; 0.40)	0.32 (0.28; 0.37)	0.013^*^	0.39 (0.35; 0.51)	0.38 (0.31; 0.51)	*0.646*	*0.260*	0.25
Step length [cm]	53.0 (44.2; 58.8)	55.2 (44.8; 62.6)	*0.110*	50.7 (41.2; 57.8)	51.2 (38.6; 60.0)	*0.333*	*0.324*	0.22

**Notes:** Values are displayed as group medians with interquartile ranges (*q1; q3*) due to non-normal distribution of data; * = significant within-group differences pre-post (*P*_*within*_ ≤ 0.05) calculated with Wilcoxon signed rank test; ° = trend to significance (*P*_*within*_ ≤ 0.10); * = significant between-group differences after intervention phase (*P*_between_ ≤ 0.05) calculated with Mann–Whitney-*U*-test; ° = trend to significance (*P*_between_ ≤ .10).

**Abbreviations:***P*_*within*_: *P*-value for within-group comparison; *P*_between_: *P*-value for between-groups comparison; *r*: effect size (*r* = 0.1: small effect, ^+^*r* = 0.3: medium effect); normal: self-selected walking speed; fast: fast walking; normalcog: self-selected walking speed with additional cognitive task; fastcog: fast walking speed with additional cognitive task.

**Table 4:** Results of dual task costs of walking analysis

**Condition/Parameters**	**Dance Group (n = 11)**		**Control Group (n = 10)**		***P***_**between**_	***r***
	_**Δ**_**DTC %**	***P***_***within***_	_**Δ**_**DTC %**	***P***_***within***_		
***Normal walking speed***						
Velocity	**- 8.22**	*0.16*	**+ 0.90**	*0.96*	*0.29*	0.23
Cadence	**- 4.92**	*0.13*	**+ 2.85**	*0.80*	*0.15*	0.32^+^
Step time	**- 5.80**	*0.09°*	**+ 2.00**	*0.88*	*0.25*	0.27
Cycle time	**- 5.68**	*0.09°*	**+ 3.01**	*0.88*	*0.18*	0.29
Stance time	**- 6.69**	*0.11*	**+ 1.93**	*0.96*	*0.23*	0.26
Single support time	**- 3.60**	*0.06°*	**+ 5.38**	*0.20*	0.05*	0.43^+^
Double support time	**- 9.51**	*0.18*	**- 4.75**	*0.80*	*0.62*	0.11
Step length	**- 3.54**	*0.21*	**- 2.52**	*0.65*	*0.86*	0.04
***Fast walking speed***						
Velocity	**- 7.07**	0.04*	**- 3.05**	*0.88*	*0.29*	0.23
Cadence	**- 4.35**	*0.09°*	**+ 0.62**	*0.72*	*0.16*	0.31^+^
Step time	**- 6.27**	*0.08°*	**+ 5.03**	*0.58*	*0.16*	0.31^+^
Cycle time	**- 6.02**	*0.08°*	**+ 4.74**	*0.58*	*0.16*	0.31^+^
Stance time	**- 7.29**	*0.08°*	**+ 5.36**	*0.58*	*0.16*	0.31^+^
Single support time	**- 3.51**	*0.21*	**+ 4.18**	*0.51*	0.04*	0.45^+^
Double support time	**- 13.42**	0.03*	**+ 9.23**	*0.33*	*0.18*	0.29
Step length	**- 3.73**	0.02*	**- 2.92**	*0.72*	*0.83*	0.05

**Notes:** Dual task cost are illustrated as the percentage of the mean difference between pre and post intervention (_Δ_ DTC %), negative values represent a reduction of DTC, positive values represent an increase in DTC; * = significant within-group differences pre-post (*P*_*within*_ ≤ 0.05) calculated with Wilcoxon signed rank test; ° = trend to significance (p_*within*_ ≤ 0.10); * = significant between-group differences after intervention phase (*P*_between_ ≤ 0.05) calculated with Mann–Whitney-*U*-test; ° = trend to significance (*P*_between_ ≤ 0.10).

**Abbreviations:***P*_*within*_: *P*-value for within-group comparison; *P*_between_: *P*-value for between-groups comparison; *r*: effect size (*r* = 0.1, small effect, ^+^r = 0.3, medium effect).

Table 4 summarizes the results of the DTC of walking analysis. Significant between-group differences for Δ DTC were observed for the parameter single support time for both normal (*U* = 27, *P* = 0.049, *r* = 0.43) and fast walking speed (*U* = 26, *P* = 0.041, *r* = 0.45). Further, in the DG the Δ DTC decreased throughout all parameters in both walking conditions from pre to post intervention. In contrast the CG demonstrated an increase in Δ DTC values after the intervention when compared to baseline data.

### Perceived fear of falling

The results of the FES-I questionnaire showed a reduction of concerns about falling in both groups. In the DG the mean value (mean, SD) was lowered from 23.7 ± 6.4 to 21.8 ± 5.0 and in the CG the mean value was reduced from 24.5 ± 4.2 to 19 ± 4.1. Between-group comparison after the twelve-week regimen resulted in non-significant (*U* = 38, *P* = 0.134, *r* = 0.32) differences.

### Gaze behavior

Substantial losses of participants for the analysis of the eye-tracking data were documented. From the 17 participants who performed the FPA test only seven data sets were complete. Reasons for the losses were mainly attributable to problems with the handling of the eye tracking system. For the sake of completeness the gaze data is presented in Additional file 2 and not further referred to in the following section.

## Discussion

This randomized controlled trial was designed to test whether a twelve-week strength and balance exercise regimen, that includes a dance video game as an additional cognitive element, would lead to greater changes in measures of gait performance and fear of falling, compared to strength and balance exercise alone. Although both groups attained improvements in gait performance and were able to reduce their concerns about falling, the results suggest positive interaction effects in favor of the dance video game group. The finding of this study supports the notion that it is advantageous to combine physical and cognitive training into clinical practice. The combination seems to have a positive influence on older adults walking abilities under dual task conditions in comparison to more traditional exercise forms [17,18].

The most prominent differences between the training groups were observable in the gait analysis. The CG demonstrated significant positive within-group changes of several spatio-temporal parameters, however, merely in the single task condition and at preferred gait speed (*normal*). Furthermore, this group exhibited a gain in velocity in the *normalcog* condition. This merely confirms findings from a systematic review that a strength and balance exercise regimen is able to preserve or improve walking abilities, even in advanced age [49]. The goal of this study, however, was to improve walking behavior under dual task conditions. The results of previous studies with similar groups, which were performing progressive machine-driven resistance training complemented with functional balance exercises, revealed no improvement of performance under attention demanding circumstances; e.g. no changes in the dual task costs of walking [50,51]. Daily activities pose high cognitive demands and safe walking should be practicable also under cognitive distractive or otherwise challenging conditions. The results of the DG show significant positive within-group differences for most gait parameters also in the dual task conditions *normalcog* and *fastcog*, thus confirming findings from previous pilot studies with similar results for dual task related costs [27,30]. Furthermore, significant between-group differences in the dual task condition *fastcog* were observed for gait velocity and single support time in favor of the DG. *Fastcog* is the condition with the most challenging motor and cognitive demands. In the present study the positive effect on DTC of gait, represented by the decrease in Δ DTC values in the DG (Table 4), may be attributed to the additional input provided by the dance video game. Thus, this substantiates the hypothesis that an additional cognitive challenge should be preferably part of a training program aiming to improve physical functioning in older adults, especially under dual task conditions. Unfortunately, how gait under these conditions should be improved has not yet been well-studied in general [52] and this study is one of the first that shows that an improvement in dual task walking with an exercise intervention supplemented by a video game is achievable.

In the FPA test both groups revealed a more accurate foot placement in M-L direction over all the walking conditions, however, only the DG manifested significant within-group differences after the intervention. The better performance may be in part attributable to improvements in walking and balance skills gained by the strength and balance exercises. A higher postural balance confidence during swing phase of the gait cycle has possibly enabled a more accurate targeting. However, a more efficient movement planning and a possible change in visual scanning of the walking path have possibly led to the better performance in the FPA test in favor of the DG. Interactive video games, like the dance video game used in this study, require precise visuo-motor control, that is to focus attention on the screen and the concurrent execution of controlled body movement and the regulation of postural control. Interestingly in this context is that expert action video game players were found to have an improved spatial distribution and resolution of visual attention, a more efficient visual attention over time and were able to attend a higher number of objects simultaneously compared to non-players [53,54], thus allowing a better allocation of the attentional resources over a visuo-motor task.

Interestingly, in both exercise groups the mean distance error in A-P direction increased after the intervention. Participants were able to navigate quicker through the test path thereby controlling their M-L direction walking deviation, however, suffered the loss of accuracy in mean distance error in A-P direction. The higher inaccuracy in the A-P direction may be in part explained by the higher waking velocity in the second test. It can be suggested, that participants gave more priority to their walking performance (greater velocity, larger steps) and their navigation towards the target in M-L direction, so that their foot placement accuracy in A-P direction decreased (speed-accuracy tradeoff) [55]. The quality evaluation, however, shows in general a qualitative better performance in the second FPA test with less shoe contacts with the targets (Table 2).

The reason to use a dance video game or video games in general, is mainly based on the findings of a systematic review [17]. It is, however, also related to the numerous advantages attributed to such a tool [25]. As known from the principles of motor learning, repetition is important for both motor learning and the cortical changes that initiate it [56]. The repeated practice must be linked to incremental success at some task or goal. A computerized intervention like the dance video game constitutes a powerful tool to provide participant repetitive practice, feedback about performance and motivation to endure practice [56]. In addition, it can be adapted based on the individual participant’s baseline motor performance and be progressively augmented in task difficulty. Further, the addition of a challenging video game has the potential to engage people who otherwise would lack of interest to participate in a physical exercise regimen. Especially in the older population it is difficult to maintain high adherence to training programs [57]. The participants of the present study showed excellent compliance rates. The losses related to low exercise compliance or low motivation (n = 4) in the DG were caused by animosities between the participants of one training group and in part by not perceiving any changes in performance level at the half of the study. The reasons for discontinuation of training were not because of rejection of the dance video game per se. The DG members were motivated by the additional playing of the video game at the end of every training session. The CG members were motivated by the assurance that after the end of the intervention they had the opportunity to include the dance video game in their exercise program as well. In both hostels, the training sessions with the additional dance video game were pursued also after the study ended.

The high acceptance of the dance video game used in our study seems at variance with reports of elderly being rather skeptical towards using commercially available games in a hospital setting [58]. We think that this is partly explainable due to the modifications made to the original dance video game free-ware StepMania. The information on the screen was reduced to a minimum and the music was chosen according participants’ taste. In general, commercially available video games are often not adapted to the needs and preferences of older adults, since they are designed for children and young adults. The games are not easy to comprehend and the screens are flashing. This might be one of the reasons why some commercially available video games are rather disliked by older adults [58].

### Limitations of the study

The present study contains some limitations that have to be discussed. A limitation of the FPA test is that different shoe sizes of the participants are not accounted for. The further development of the FPA test protocol should consider foot size by using different sized foam targets. We assume that a subject with small feet has more free space in the target area to place his/her foot before touching the border of the target resulting in a potentially higher risk of becoming variable in the accuracy performance. On the other hand the larger the foot the smaller the free space between foot and border of the target. A subject with large feet will not have a comparable amount of potential variability of distance errors as the person with small feet.

An obvious limitation of our study is the rather small sample size. This study, therefore, only reveals first estimates for these measures and warrants further research in larger populations. When evaluating the validity of a study it is important to consider both the clinical and statistical significance of the findings [59]. Studies that claim clinical relevance may lack sufficient statistical significance to make meaningful statements or, conversely, may lack practicality despite showing a statistically significant difference in treatment options. Researchers and clinicians should not focus on small *P*-values alone to decide whether a treatment is clinically useful; it is necessary to also consider the magnitude(s) of treatment differences and the power of the study [59]. Encouraging in this context is the observation that the majority of the between groups comparisons show medium or medium-to-high magnitude(s) of treatment differences. This in mind, the relationship between physical and cognitive training research and its effect on gait in elderly individuals requires further exploration. Future adequately powered studies with similar populations should, therefore, be performed to substantiate our assumption and findings.

The suggested link between the observed improvement in the physical tests after the intervention and influences on cognitive processes in the brain is as of yet still speculative. A necessary next step would be to investigate the isolated effects of the video game on measures of cognitive functioning. Since improvements were observable in physical performance under attention demanding circumstances it seems plausible to hypothesize that these changes may rely, at least in part, on functional or even structural changes in the brain. A recently published study protocol [60] might be able to provide some insights on this topic.

## Conclusions

Our results support previous larger studies that strength and balance exercise may lead to better walking performance in older untrained subjects. Integrating a cognitive training component in addition, results in further improvements in those walking tasks that are related to cognitive functions. Enhancements in walking performance under dual task conditions were observed for the dance video game group only. Our findings suggest that the addition of this particular program to traditional strength and balance exercises may result in improved outcomes for older people. An exercise program that aims to improve physical functioning in older adults under dual task conditions should also consider a cognitive challenging element, preferably in form of an interactive video game adapted for older adults, in addition to strength and balance exercises.

## Competing interests

The authors declare that they have no competing interests.

## Authors’ contribution

Conception, design and manuscript drafting: GP, EDdB: Critical revision of manuscript for its content and approval of final version: GP, EDdB, KM. All authors read and approved the final manuscript.

## Pre-publication history

The pre-publication history for this paper can be accessed here:

http://www.biomedcentral.com/1471-2318/12/74/prepub

## Supplementary Material

Additional file 1 Detailed results of foot placement accuracy. Click here for file

Additional file 2 Results of gaze behavior assessment during FPA test. Click here for file
