# Supplementary material for: A cognitive-motor intervention using a dance video game to enhance foot placement accuracy and gait under dual task conditions in older adults: a randomized controlled trial
Source: BMC Geriatr. 2012 Dec 14;12:74. doi: 10.1186/1471-2318-12-74 (PMC3538689; doi:10.1186/1471-2318-12-74)
Supplement: Additional file 1 — Detailed results of foot placement accuracy. [file 1471-2318-12-74-S1.doc]

**Additional File 1** Detailed results of foot placement accuracy

|  | **Dance group** (n=8) | |  | **Control group** (n=9) | |  |  |
| --- | --- | --- | --- | --- | --- | --- | --- |
| Condition/Target | pre | post | *P_within_* | pre | post | *P_within_* | *P*_between_ |
| *Medio-lateral error [mm]* |  |  |  |  |  |  |  |
| C1/T1 | 15.00 (11.44; 17.33) | 12.09 (8.95; 17.63) | *0.48* | 12.96 (9.90; 16.71) | 10.92 (8.72; 20.04) | *0.86* | *0.77* |
| C2/T1 | 12.27 (10.20; 14.74) | 10.93 (8.92; 12.52) | *0.21* | 11.72 (9.16; 16.69) | 13.71 (10.20; 17.96) | *0.31* | *0.15* |
| C3/T1 | 10.42 (8.38; 15.20) | 9.84 (8.50; 15.09) | *0.58* | 11.00 (4.62; 17.86) | 8.27 (6.78; 16.75) | *1.00* | *1.00* |
| C2/T2 | 13.51 (8.98; 20.10) | 9.93 (5.65; 15.36) | *0.07°* | 12.52 (10.98; 20.16) | 13.01 (8.37; 2.08) | *0.72* | *0.39* |
| C3/T2 | 12.97 (5.79; 16.00) | 9.65 (6.78; 10.93) | *0.16* | 9.62 (3.16; 17.14) | 9.16 (6.78; 1.34) | *0.48* | *0.29* |
| *Anterior-posterior error [mm]* |  |  |  |  |  |  |  |
| C1/T1 | 19.82 (12.78; 29.17) | 19.59 (12.98; 26.44) | *0.58* | 22.92 (17.22; 32.28) | 21.40 (13.25; 34.01) | *0.52* | *0.21* |
| C2/T1 | 17.76 (12.77; 29.88) | 22.32 (14.49; 31.21) | *0.58* | 14.42 (10.21; 28.49) | 23.55 (16.47; 30.18) | *0.26* | *0.63* |
| C3/T1 | 16.80 (14.88; 26.44) | 27.86 (14.26; 39.72) | *0.26* | 8.34 (3.58; 23.23) | 18.75 (15.05; 26.88) | 0.04* | *0.77* |
| C2/T2 | 22.85 (13.32; 28.90) | 21.17 (15.15; 26.42) | *0.67* | 18.60 (16.88; 34.12) | 24.97 (18.37; 31.52) | *0.86* | *0.63* |
| C3/T2 | 16.56 (14.06; 27.15) | 18.52 (13.49; 29.33) | *0.40* | 19.84 (5.39; 21.51) | 14.97 (11.53; 20.17) | *0.89* | *0.56* |

**Notes:** Values are displayed as group medians with interquartile ranges (*q1; q3*) due to non-normal distribution of data; * = significant **within**-group differences pre-post (*P_within_* ≤ 0.05) calculated with Wilcoxon signed rank test; ° = trend to significance (*P_within_* ≤ 0.10);

**Abbreviations:** *P_within_*: *P*-value for within-group comparison; *P*_between_: *P*-value for between-groups comparison; C1, C2, C3: conditions 1, 2, and 3; T1, T2: Target 1 and 2
