# Supplementary material for: A cognitive-motor intervention using a dance video game to enhance foot placement accuracy and gait under dual task conditions in older adults: a randomized controlled trial
Source: BMC Geriatr. 2012 Dec 14;12:74. doi: 10.1186/1471-2318-12-74 (PMC3538689; doi:10.1186/1471-2318-12-74)
Supplement: Additional file 2 — Results of gaze behavior assessment during FPA test. [file 1471-2318-12-74-S2.doc]

**Additional File 2** Results of gaze behavior assessment during FPA test

| **Subject** | **Group** | **Classification**^1^ | **Gaze fixation** [ms] PRE / POST | | | | |  | **Gaze on target at heel contact** [%] PRE / POST | | |
| --- | --- | --- | --- | --- | --- | --- | --- | --- | --- | --- | --- |
|  |  |  | C1 - T1 | C2 - T1 | C3 - T1 | C2 - T2 | C3 - T2 |  | C1 | C2 | C3 |
| 1 | CG | faller | 164 / -171 | 180 / 87 | 132 / 140 | 40 / 20 | 90 / 74 |  | 80 / 0 | 75 / 65 | 83 / 81 |
| 2 | CG | faller | 4 / 153 | 22 / 40 | -72 / 24 | 472 / n.d. | 416 / 232 |  | 50 / 63 | 70 / 73 | 55 / 64 |
| 3 | DG | non-faller | -820 / 151 | -675 / 124 | -1005 / 188 | -314 / 244 | -510 / 292 |  | 0 / 100 | 0 / 95 | 0 / 100 |
| 4 | DG | non-faller | 228 / 380 | 240 / 102 | 148 / 52 | 320 / 188 | 276 / 132 |  | 80 / 100 | 100 / 85 | 85 / 75 |
| 5 | DG | non-faller | -172 / 216 | 6 / 145 | -1225 / 124 | 160 / 240 | -780 / 220 |  | 30 / 100 | 59 / 90 | 38 / 95 |
| 6 | DG | non-faller | -692 / 336 | -395 / 87 | -140 / 330 | -293 / 248 | -98 / 400 |  | 0 / 100 | 0 / 73 | 37 / 100 |
| 7 | DG | faller | -347 /-613 | 84 / -18 | 216 / n.d. | 449 / 260 | 369 / n.d. |  | 13 / 11 | 100 / 60 | 100 / n.d. |

**Notes**: ^1^ = according to Di Fabio et al 2001 [48]; Negative fixation values correspond to premature gaze shift from the target prior to heel contact, i.e. time from gaze shift to heel contact; positive fixation values correspond to the time of persistent fixation on target after heel contact; the values ‘gaze on target’ provide information on the location of gaze at heel contact (100 % = gaze on target on every trial);

**Abbreviations:** CG, control group; DG, dance group; C1-3, Conditions 1-3; T1-2, Target 1-2; n.d., no data
